# Integrating Concentration-Dependent Toxicity Data and Toxicokinetics To Inform Hepatotoxicity Response Pathways

**DOI:** 10.1021/acs.est.3c02792

**Published:** 2023-08-11

**Authors:** Daniel P. Russo, Lauren M. Aleksunes, Katy Goyak, Hua Qian, Hao Zhu

**Affiliations:** † Department of Chemistry and Biochemistry, Rowan University, Glassboro, New Jersey 08028, United States; ‡ Department of Pharmacology and Toxicology, Ernest Mario School of Pharmacy, Rutgers University, Piscataway, New Jersey 08854, United States; § ExxonMobil Biomedical Sciences, Inc., Annandale, New Jersey 08801, United States

**Keywords:** supervised learning, big data, data mining, adverse outcome pathway modeling, biological pathway modeling

## Abstract

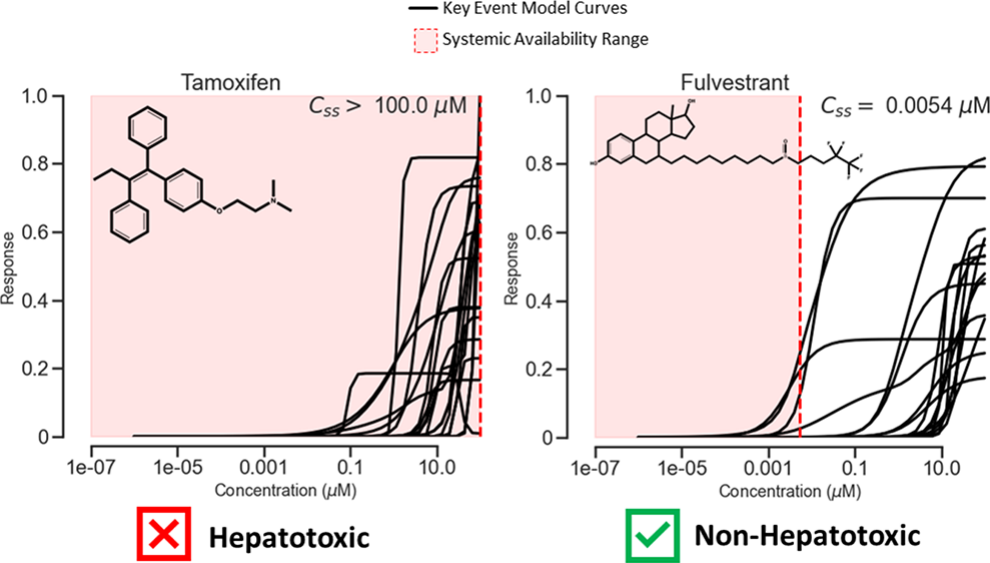

Failure of animal models to predict hepatotoxicity in humans has created a push to develop biological pathway-based alternatives, such as those that use in vitro assays. Public screening programs (e.g., ToxCast/Tox21 programs) have tested thousands of chemicals using in vitro high-throughput screening (HTS) assays. Developing pathway-based models for simple biological pathways, such as endocrine disruption, has proven successful, but development remains a challenge for complex toxicities like hepatotoxicity, due to the many biological events involved. To this goal, we aimed to develop a computational strategy for developing pathway-based models for complex toxicities. Using a database of 2171 chemicals with human hepatotoxicity classifications, we identified 157 out of 1600+ ToxCast/Tox21 HTS assays to be associated with human hepatotoxicity. Then, a computational framework was used to group these assays by biological target or mechanisms into 52 key event (KE) models of hepatotoxicity. KE model output is a KE score summarizing chemical potency against a hepatotoxicity-relevant biological target or mechanism. Grouping hepatotoxic chemicals based on the chemical structure revealed chemical classes with high KE scores plausibly informing their hepatotoxicity mechanisms. Using KE scores and supervised learning to predict in vivo hepatotoxicity, including toxicokinetic information, improved the predictive performance. This new approach can be a universal computational toxicology strategy for various chemical toxicity evaluations.

## Abstract 2

A computational framework capable of modeling biological pathways that integrates with toxicokinetic information is described. This modeling framework can identify potential hepatotoxicants and their mechanisms of hepatotoxicity.

## 1 Introduction

Chemical hepatotoxicity is the injury to the liver imposed by chemicals before or after their metabolic transformation and is a major concern to public health. In the pharmaceutical sector, the high incidence of hepatotoxicity is a leading cause of drug attrition and results in large financial losses for pharmaceutical companies.^1^ In industrial chemical risk assessment, liver effects are one of the most commonly observed outcomes in animal studies conducted for the purpose of hazard identification,^2^ likely due to the liver’s role in the metabolism and excretion of the chemical substance being tested. Testing chemicals for hepatotoxicity has traditionally been done using animal models. However, predicting hepatotoxicity using animal models is complicated both by low intra-species concordance between animals and humans^3−5^ and by high inter-individual variability in humans,^6,7^ a key factor contributing to idiosyncratic drug-induced liver injury. Additionally, the use of animal models to test chemicals for hepatotoxicity is costly, both in terms of dollars and animal use. The high resource cost with limited relevance to human health human risk assessment^8^ has prompted a call to revamp traditional chemical toxicity testing.^9−12^ This revamped approach relies on an increased understanding of the biological pathways leading to target organ effects, such as liver activation. Liver response pathways for effects resulting in cholestasis,^13^ steatosis,^14^ and fibrosis^15^ are available; however, expansion of these early examples through identification and convergence of biological events leading to hepatotoxicity remains a knowledge gap urgently required to refine predictions for both pharmaceutical and industrial chemicals.^16^

In 2007, the National Research Council outlined a roadmap for the future of toxicity testing, calling for a shift in strategy from traditional animal testing to high-throughput alternatives, such as those that use computational or in vitro approaches.^17^ Progress toward the vision communicated in this roadmap has been accelerated by the development of biological pathway-based toxicity evaluations, also called adverse outcome pathways (AOPs), a conceptual framework for organizing the key biological events (KEs) leading to toxicities after chemical exposures.^18−20^ To achieve this goal, high throughput screening (HTS) projects, such as the Toxicity Forecaster (ToxCast) and Toxicity Testing in the 21st Century (Tox21), screened thousands of chemicals in hundreds of different cell-based and cell-free in vitro assays.^21^ The result of these efforts was an enormous amount of HTS data linked to specific molecular processes (e.g., nuclear receptor activation). Models of AOPs that leverage responses across multiple related assay targets can simulate the underlying in vivo toxicological processes. For example, 18 ToxCast and Tox21 HTS assays related to the estrogen receptor pathway were incorporated in an AOP model, which was accepted by regulatory agencies as an alternative to animal toxicity testing to identify potential estrogens.^22,23^ Using a similar strategy, another AOP modeling study was performed by building AOPs for individual estrogen receptor agonists using a novel deep learning network.^24^ However, these computational models rely on prior knowledge of the AOPs and HTS assays relevant to the target toxicity. For complex toxicities such as hepatotoxicity, AOPs are available for some liver toxicities (e.g., cholestasis, fibrosis, steatosis);^13−15^ however, to be useful to inform testing strategies, expansion to common KEs within AOPs leading to a diverse array liver toxicities is needed.^16,25^ Recently, developing AOP models for complex toxicity endpoints from sources of HTS assays have been reported, but these methods are only capable of using HTS data of a single assay, which is not sufficient to cover the multiple biological processes or targets involved in the KEs of AOPs.^26,27^ Additionally, these models do not account for the toxicokinetics of chemicals, which is the estimated in vivo concentration of a chemical after accounting for human metabolism and clearance. Relating the concentrations at which a chemical is tested in vitro to systemic availability in vivo is a critical component when using in vitro assays to predict in vivo toxicity.^28,29^ Thus, novel computational methods are needed to leverage available and robust HTS data sufficiently to develop AOP models for complex toxicities such as hepatotoxicity.^30^ Additionally, new chemical descriptors in machine learning are necessary and important for predicting toxicity.^31^ The integration of HTS data as descriptors could result in more predictive and accurate machine-learning models.

In this work, we present a new computational approach to develop AOP models from concentration-dependent HTS data for complex toxicity endpoints. Using a comprehensive database^32^ of 2171 chemicals with human hepatotoxicity data, we extracted HTS assays from ToxCast/Tox21 with correlations to hepatotoxicity. These HTS assays target diverse biological KEs relevant to hepatotoxicity mechanisms. Using a computational model, these 157 HTS assays were grouped into 52 KE models related to hepatotoxicity. These models generate a KE score for compounds, allowing target chemicals to be ranked according to their potency against a particular hepatoxicity mechanism. Lastly, supervised learning was used to build a hepatotoxicity model combining KE scores and bioavailability information estimated from toxicokinetic modeling to predict the in vivo hepatotoxicity of chemicals. This computational method is a promising end-to-end approach to achieving the goal of developing high-throughput and mechanistic-based models for complex toxicity evaluations.

## 2 Methods

### 2.1 ToxCast/Tox21 HTS Data Processing

ToxCast and Tox21 HTS data were downloaded via the EPA’s website (https://www.epa.gov/chemical-research/exploring-toxcast-data-downloadable-data, accessed August 2021). The summary files obtained from this resource contain the concentration response data (e.g., AC_50_ values calculated by curve fitting) of more than 12,000 chemicals tested against over 1600 HTS assays. For an individual assay, each tested chemical has results from three different curve-fits of the concentration response along with scores measuring goodness-of-fit.^33^ In this work, the results with the best goodness-of-fit score (i.e., lowest curve error) for every chemical and assay were selected. Using the parameter values generated from the best goodness-of-fit score, we created a concentration response curve for concentrations ranging from 1 pM to 100 mM as described in previous publications.^22,23^ These concentration-response curves were created using a standard hill curve equation as follows:
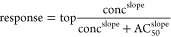
where top, slope, and AC_50_ are the parameters obtained from the summary files and conc are concentrations in the range as described above. Chemicals with constant activities in all concentrations (i.e., showed no response) were assigned a flat curve (i.e., 0 response value for each concentration). Because responses for different assays varied (e.g., percent response or fold-change increase) and resulted in different scales, all responses in an assay were normalized to the range of [0, 1].

In addition, the summary files contain “hit calls”, a binary active/inactive classification derived from chemical concentration response curves in assays. These classification results were used to identify the assays relevant to hepatotoxicity described below. Additional information about these classifications and the processing of ToxCast/Tox21 data can be found in the references.^33^

### 2.2 Hepatotoxicity Dataset

The comprehensive hepatotoxicity dataset used in this study was obtained from a previous publication.^32^ This hepatotoxicity dataset was compiled from a variety of public databases and literature resources and contained chemical in vivo hepatotoxicity outcomes from both pre-clinical (i.e., animal models) and clinical (i.e., human) studies. Specific mechanistic information was obtained for hepatotoxicants through either morphological observation (e.g., liver histopathology examinations) and/or clinical chemistry test results (e.g., liver enzyme levels from blood tests). In this work, we focused on the data that were from clinical reports (i.e., human data) and classified hepatotoxicants as being hepatocellular and/or hepatobiliary toxicants based on their morphological and clinical chemistry findings when applicable.

### 2.3 Identifying ToxCast/Tox21 Assays Relevant to Hepatotoxicity

To identify ToxCast/Tox21 assays relevant to hepatotoxicity, we calculated the correlation between hepatotoxicity outcomes (i.e., hepatotoxic, nonhepatotoxic) with the assay hit call classification (i.e., active, inactive). To evaluate specific hepatotoxicity mechanisms, the hepatobiliary and hepatocellular classifications were correlated to assay hit calls separately. We determined the significance of correlations using Fisher’s Exact Test and corrected for multiple-hypothesis testing using the Benjamini–Hockberg (BH) procedure with a false discovery rate of 0.15.^34^ This cutoff resulted in sufficient data for modeling and was in line with our previous efforts in controlling false positives.^26,27^ The selected significant assays were then manually inspected and grouped according to similar biological targets, pathways, or mechanisms. For example, assays that targeted the same pathway (e.g., estrogen receptor antagonism) or biological mechanism (e.g., DNA Repair) were grouped together as a KE of hepatotoxicity. The KE classifications of the ToxCast/Tox21 assays used in this work are available in the supplemental file (SupplementalFile.xlsx).

### 2.4 Computational Key Event Models of Hepatotoxicity

HTS assays assigned to a related biological mechanism or target were combined into a KE model using a linear additive method.^22,23^ To deal with missing data, untested chemicals or those without concentration-response parameters (i.e., no response in the tested concentrations) were assigned a zero value across all concentrations. Normally, the hit rates for HTS assays are generally low (e.g., lower than 5%).^35,36^ This assumption of inactivity for untested chemicals was conservatively chosen to reduce false positives. In this approach, the KE model output for a chemical is a composite of the related concentration-response HTS assays (i.e., assays grouped as a KE as stated in the previous section). This output is a set of values, which were defined as KE curves and summarized chemical activities across the range of tested concentrations. A KE curve mimics a composite concentration-response curve, and the area under this curve, which was defined as the KE score, is a summary value that can be used to rank chemicals based on their responses for the relevant assays of a KE. The KE scores were scaled to [0, 1], where 1 represents the chemical with the highest responses across all concentrations of all assays (i.e., the chemical with the highest KE curve).

### 2.5 Estimating Bioavailability through Toxicokinetics Modeling

The in vivo bioavailability of chemicals was estimated using the toxicokinetics modeling software, *httk*.^37^ This software contains the parameters (e.g., plasma protein binding and hepatic clearance) needed for modeling different tissue concentrations for environmental chemicals and pharmaceuticals, including many of the ToxCast/Tox21 chemicals. The concentration at the steady state (*C*_ss_) is the approximate blood plasma concentration of a chemical after repeated exposure and accounting for metabolic processes and can be used as the quantitative representation of systemic exposure. For all available chemicals, we used this software to calculate *C*_ss_ values after 1 mg/kg/day oral exposure. Because different human populations metabolize chemicals at different rates, the *C*_ss_ values for a chemical are reported as a set of normally distributed values, representing variability across human populations. The 5% quantile represents populations of humans with faster metabolism/clearance (i.e., lower *C*_ss_ values), and the 95% quantile represents those with slower metabolism/clearance (i.e., higher *C*_ss_ values). In this work, the conservative *C*_ss_ value corresponding to the most vulnerable population (i.e., the 95% quantile) was used to evaluate chemicals.

### 2.6 Semi-Supervised Modeling of Hepatotoxicity

Identifying the relationship among KEs that contribute to an AOP and are capable of predicting in vivo hepatotoxicity is often not apparent without extensive literature searching and testing. Therefore, to build a hepatoxicity model, we used logistic regression to predict in vivo hepatotoxicity using KE scores obtained from our KE models. Logistic regression models are a regression technique used to predict the binary response (i.e., hepatotoxic or not hepatotoxic) given a set of predictor variables. Two approaches were used to train logistic regression models to predict hepatotoxicity. The first approach used only KE scores as predictor variables. The second approach used KE scores but also included chemical bioavailability information – toxicokinetic information included *C*_ss_ values and calculated molecular descriptors relating to Lipinski’s rule of five for estimating oral bioavailability. The Lipinski descriptors include lipophilicity (LogP), the number of hydrogen bond donors and acceptors, the number of rotatable bonds, molecular weight, and others. The full list of calculated Lipinski descriptors can be found online: https://www.rdkit.org/docs/source/rdkit.Chem.Lipinski.html. Only the ToxCast/Tox21 chemicals with hepatotoxicity data and *C*_ss_ values from the *httk* were used for logistic modeling.

## 3 Results and Discussion

### 3.1 Identifying ToxCast/Tox21 Assays Relevant to Hepatotoxicity

The ToxCast/Tox21 chemical library consists of chemicals of public health concern and is biased toward chemicals with known toxicity.^38^ The ToxCast/Tox21 screening program has tested its library of more than 10,000 chemicals in over 1600 assays. Approximately 81% (1762 out of 2171) of the chemicals from the hepatotoxicity database were found in the ToxCast/Tox21 chemical library. Most of the chemicals were classified as hepatocellular or hepatobiliary toxicants. Overall, the ToxCast/Tox21 chemicals with hepatotoxicity data were biased toward hepatotoxicity, with 68% (1200/1762) of the chemicals classified as hepatotoxicants. Among these hepatotoxicants, 81% (969/1200) were classified as hepatocellular toxicants, and 61% (726/1200) were classified as hepatobiliary toxicants. There were 57% (682/1200) of toxicants classified as involving both mechanisms for their toxicity.

The assays correlated to hepatocellular and hepatobiliary toxicity were selected using Fisher’s exact test. A total of 157 assay results were found to be significantly correlated to hepatocellular and/or hepatobiliary toxicity. The number of assays correlated to hepatobiliary toxicity (148 out of 157 assays) was much greater than those correlated with hepatocellular toxicity (36 out of 157 assays). Notably, 27 assays were correlated to both hepatocellular and hepatobiliary toxicity.

### 3.2 Key Events Associated with Hepatotoxicity

The 157 assays relevant to hepatocellular and/or hepatobiliary toxicity targeted various biological processes, including cell stress responses, nuclear receptors, metabolism, transport, cell morphology, and cell growth/regulation. Assays determined to target a shared biological process were grouped together and considered a KE of hepatotoxicity. For example, if multiple assays targeted the same receptor binding mechanism (e.g., estrogen receptor antagonism) or the same biological process (e.g., DNA repair), they were grouped together and considered a KE. The distributions of the 157 assays among 52 KEs are provided in SupplementalFile.xlsx and the top 15 KEs (ranked by number of underlying assays) are shown in Table 1. The number of assays per KE ranged from 1 to 59 and 42% (22 out of 52) of the KEs have more than one assay.

These KEs obtained from groups of assays captured critical biological processes relevant to hepatotoxicity. For example, KE 1 (cytotoxicity), which consisted of the most assays, had 59 cell viability assays across different cell lines. A variety of cell stress pathways were found as KEs, such as KE 3, which contained 6 assays targeting the *p53* tumor suppressor protein, a marker of genotoxic stress.^39^ KE 7 was another cell stress pathway and consisted of 3 assays that measure chemicals’ potential for inflicting DNA damage.^40^ KE 14 captured oxidative stress potential and contained 3 assays that measured cellular antioxidant responses induced by electrophilic chemicals via the nuclear factor erythroid 2-related factor 2 (Nrf2) pathway.^41^ Several assays targeted nuclear receptors, and these included the estrogen receptor (ER, KE 4), androgen receptor (AR, KE 8), progesterone receptor (PG, KE 12), and the peroxisome proliferator-activated receptor (PPAR, KE 13) pathways. Also of note were several KEs involving metabolism and transport such as cytochrome P450 (CYP, KE 5 and KE 48) proteins, a sulfotransferase (SULT, KE 29), bile salt export pump (ABC, KE 52), and others. Another noteworthy set of assays was KE 15 (MITO) representing dysregulation in mitochondrial function, which is critical to liver function.^42,43^

### 3.3 Computational Key Event Models of Hepatotoxicity

**Figure 1:**
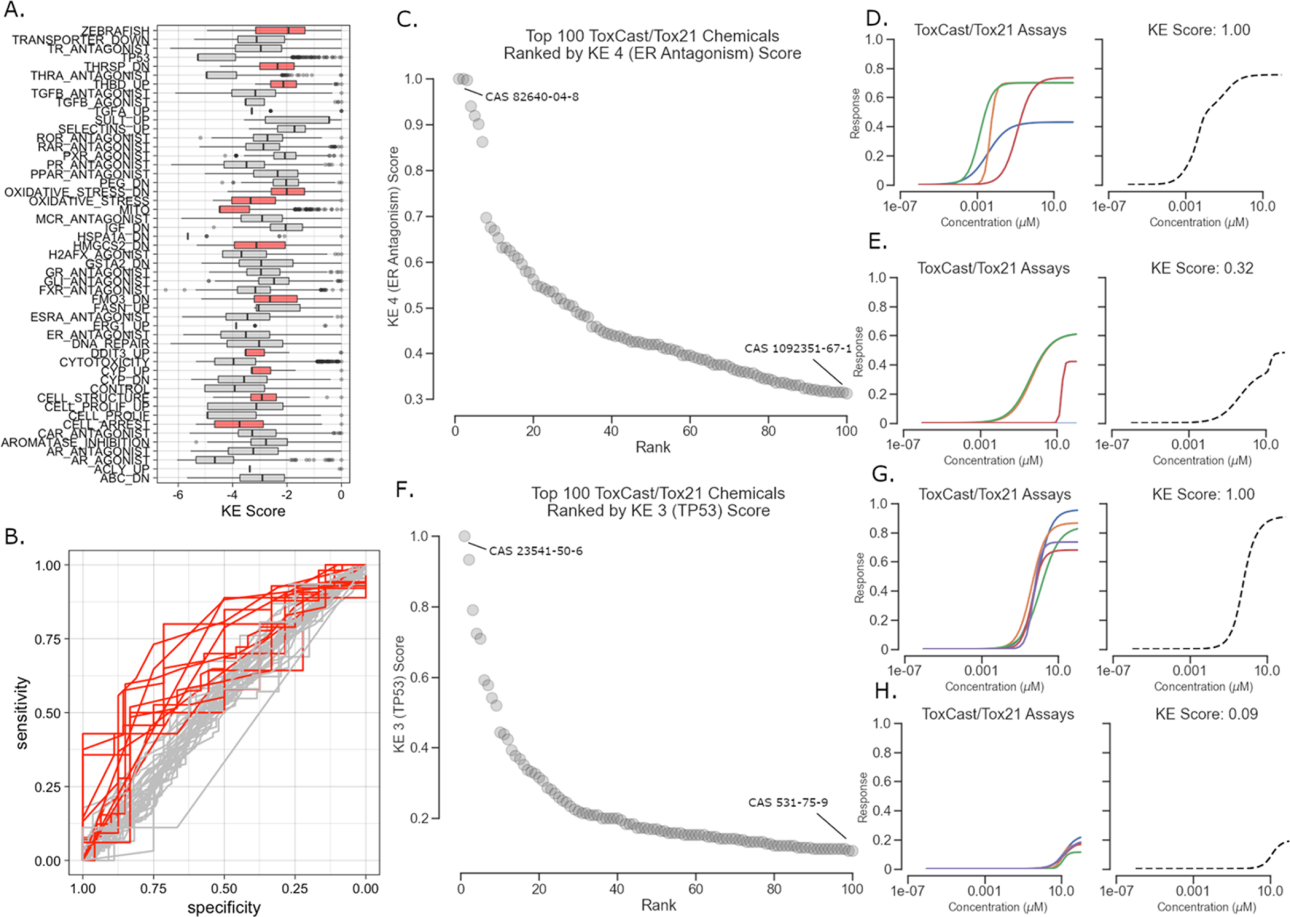
KE model overview. (A) Distribution of the logarithm of KE scores for KE models of chemicals with KE scores >0. (B) ROC curves of hepatotoxicity predictions for KE models. ROC curves with an area under the curve >0.6 are highlighted in red. (C) Top 100 ToxCast/Tox21 chemicals ranked by the ER antagonism KE model (KE 4). (D) KE 4 assay responses (left) and KE curve (right) for raloxifene hydrochloride (CAS 82640-04-8). (E) KE 4 assay responses (left) and KE curve (right) for torkinib (CAS 1092351-67-1). (F) Top 150 ToxCast/Tox21 chemicals ranked by the TP53 KE model (KE 3). (G) KE 3 assay responses (left) and KE curve (right) for daunorubicin hydrochloride (CAS 23541-50-6). (H) KE 3 assay responses (left) and KE curve (right) for esculin (CAS 531-75-9).

A computational approach was used to integrate the HTS assays assigned to KEs into individual KE models. The output of a KE model was a set of values based on the assay responses of tested chemicals in a range of concentrations, which was defined as the KE curve to reflect the underlying relationship of the concentration responses (i.e., hill curves). The KE curve was a composite in vitro concentration-response curve and provided a summary of the responses of the assays assigned to a KE. KE curves ranged from 0 to 1 and provided an estimate of the composite activity of all assays within the relevant KE for a chemical at a specific concentration. Two examples of the KE curves for ER antagonism (KE 4) are shown in Figure 1D,E. The newly generated KE curve is used to summarize the activity for this chemical at each concentration for all assays within a KE (e.g., dashed black lines in Figure 1D,E). The area under the KE curve, which was defined as the KE score, can be used as a composite value to summarize the KE curve and rank the chemical (Figure 1C). KE scores can distinguish highly potent chemicals that show high activities across multiple assays in a KE from those that show low potencies and/or low activities. As shown in Figure 1C, raloxifene hydrochloride (CAS 82640-04-8) and torkinib (CAS 1092351-67-1) have KE scores of 1 and 0.32 for KE 4, respectively. Raloxifene hydrochloride is an ER modulator developed as a drug for breast cancer.^44,45^ It was the highest-ranked ER antagonist and showed high potency across the ER antagonist assays. For comparison, torkinib has a low KE score of 0.32 and is also an anticancer drug but developed as a selective mTOR inhibitor.^46^ This compound only shows moderate activity in high concentrations and the potency is low, demonstrating that torkinib is not an ER modulator. The different effects on this KE by these two compounds are reflected in both the KE scores (Figure 1C) and the KE curves (Figure 1D,E). Similarly, the KE score can capture the differences in the maximum responses of a chemical across multiple assays within a KE as well. For example, the KE scores for KE 3 (TP53) were shown in Figure 1F. TP53 is the gene transcribing the protein *p53* and a marker of DNA damage and genotoxicity. The top ranked TP53 compound is daunorubicin hydrochloride (CAS 23541-50-6), with a KE score of 1.0, which equals the highest defined KE score. Daunorubicin hydrochloride is a DNA topoisomerase II inhibitor that causes DNA damage.^47,48^ Comparatively, esculin (CAS 531-75-9) has a low KE score of 0.09, which is close to the lowest KE score end of 0. This chemical is a glucoside that occurs naturally in chestnuts and has been investigated as a natural therapy for a variety of ailments^49−51^ and has no effects on this pathway target. The maximum response elicited by daunorubicin across the three assays is much higher compared to esculin and is also reflected in their respective KE scores (Figure 1F) and KE curves (Figure 1G,H).

Distribution of KE scores among the ToxCast/Tox21 chemicals varied across the different KEs but many compounds had KE scores of 0 due to the nature of HTS assays where most compounds show no activity.^35,52,53^ We applied a minimum threshold of KE scores (i.e., KE score > 0) to remove those compounds with 0 KE score values. The distribution of all remaining KE scores is shown in Figure 1A. The number of compounds with KE scores above this threshold varied among these KEs. Some KEs had only a few compounds with nonzero scores. For example, the KEs of the upregulation of fatty acid synthase (KE 45) and upregulation of ATP-citrate synthase (KE 51) had only one and two compounds with KE scores above 0, respectively. Conversely, other KEs, such as DNA repair (KE 7) and GLI antagonism (KE 42), have 1233 and 1023 compounds with KE scores above 0, respectively.

The predictivity of the KE models using KE scores for hepatotoxicity was assessed by the area under the receiver operating characteristic (ROC) curve. The area under the ROC curve scores for all KE models are given in SupplementalFile.xlsx and the corresponding ROC plots are displayed in Figure 1B. Among the 52 KEs, 13 of them can predict hepatotoxicity significantly (i.e., areas under the ROC greater than 0.6). These 13 KEs are highlighted in red in Figure 1A,B.

### 3.4 Chemical Clustering To Reveal Key Event Signatures of Hepatotoxicity

**Figure 2:**
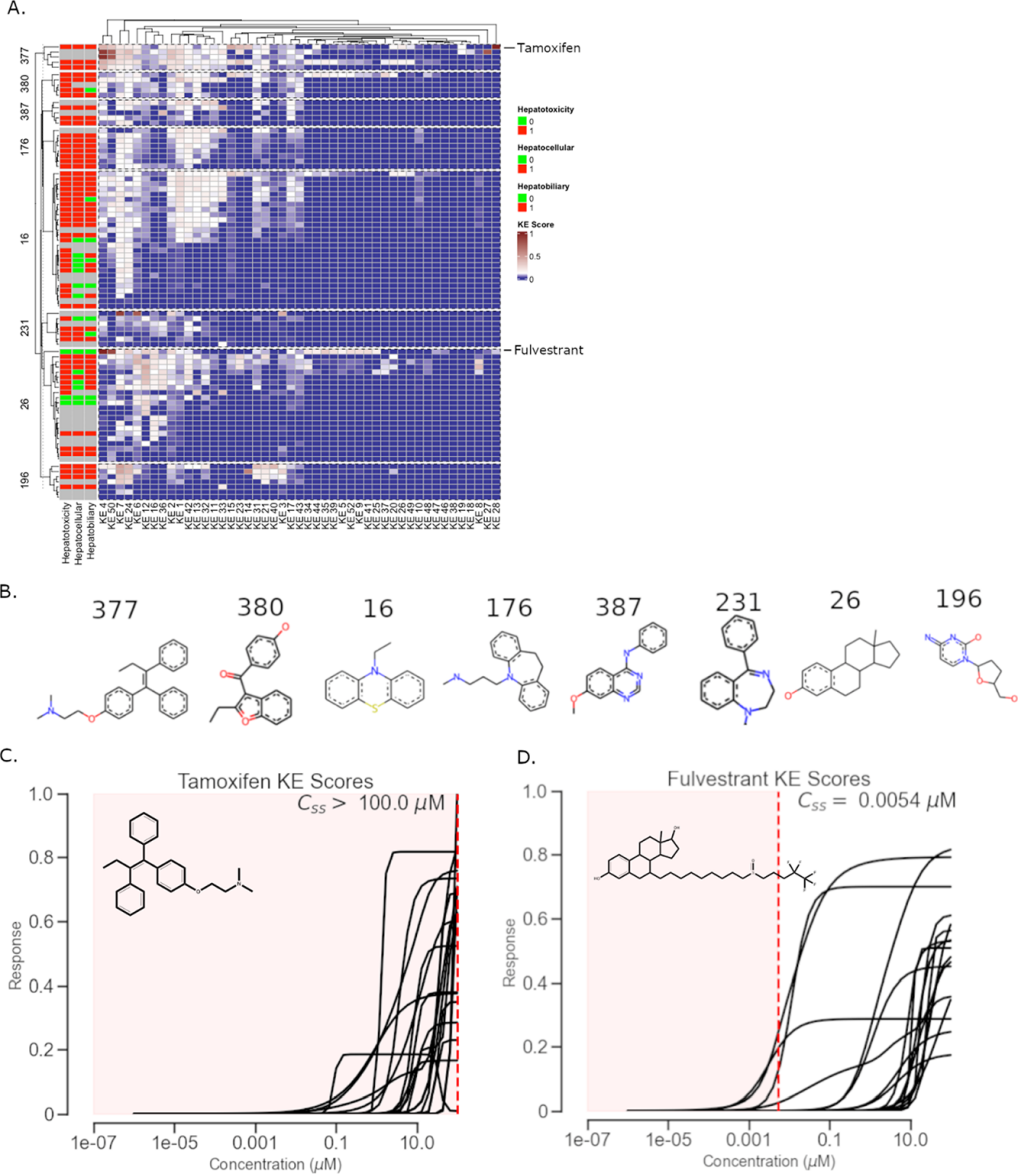
Hepatoxicity clustering. (A) KE scores for chemicals in 8 selected clusters. (B) Shared chemical structure among 8 hepatotoxicity clusters. (C) KE curves for tamoxifen (CAS 10540-29-1) with the estimated in vivo activity range highlighted in red. (D) KE curves for fulvestrant (CAS 129453-61-8) with the estimated in vivo activity range highlighted in red.

A critical component of developing AOPs is to determine the molecular initiating event (MIE), defined as the initial interaction between a toxicant and a biomolecule (e.g., receptor in a pathway) that can be causally linked to an adverse outcome through a toxicity pathway.^18^ Identifying shared chemical features among chemicals with high KE scores can identify MIEs. To this end, the ToxCast/Tox21 chemicals were grouped into clusters of chemicals with greater than 70% similarity using the Butina clustering algorithm.^54^ Within these clusters, 38 had high proportions of hepatotoxicants (i.e., >2/3 of chemicals with liver data were hepatotoxicants). The mean KE scores for these 38 hepatotoxicant clusters are shown in Figure 2A. The chemical clustering revealed that some hepatotoxicant clusters showed high KE scores for certain KEs, allowing for inference on their hepatotoxicity mechanisms and potential AOPs. For example, eight hepatotoxicant clusters (Clusters 377, 196, 380, 387, 16, 176, 26, 231) had mean KE scores >0.1 for one or more KEs. The KE scores for the chemicals in these hepatotoxicant clusters, along with their hepatotoxicity data, are shown in Figure 2A. The structures of the chemicals within a cluster consisted of a common chemical scaffold (Figure 2B).

Grouping chemicals by structure similarity revealed chemical structure-AOP relationships that explain the hepatotoxicity mechanisms of certain chemical classes. For example, Cluster 377 showed high KE scores for aromatase inhibition (KE 50) and ER antagonism (KE 4) and contained chemicals that are known ER antagonists and aromatase inhibitors in vivo, such as toremifene (CAS 89778-27-8) and tamoxifen (CAS 10540-29-1). All chemicals within this cluster shared a common tricyclic ring structure with an ether-linked tertiary alkyl amine (Figure 2B). Toremifene and tamoxifen are antiestrogens, which are used as drugs for breast cancers and are well known for their adverse effects on hepatotoxicity by inducing nonalcoholic fatty liver disease.^55^ These chemicals target the ER and aromatase pathways, whose disruptions are linked to lipid dysregulation and nonalcoholic fatty liver disease.^55−57^ Therefore, these results suggest that chemicals within this cluster can cause hepatotoxicity through altered lipid homeostasis involving ER and aromatase binding, leading to an accumulation of lipids in the liver and subsequent hepatotoxicity.

Cluster 196 showed high KE scores of DNA repair (KE 7), as well as TP53 (KE 3) and TR antagonism (KE 24). Chemicals in this cluster were pyrimidine nucleoside analogues and anti-cancer drugs, such as cytarabine (CAS 147-94-4) and gemcitabine (CAS 122111-03-9) and an anti-retroviral drug, zalcitabine (CAS 7481-89-2). These chemicals share a ribose ring linked to a cytosine (Figure 2B) and cause direct acute hepatotoxicity through their interaction with DNA and interference with DNA replication in both the nucleus and mitochondria.^58−60^ Mitochondrial function is important and the disruption of mitochondrial function is an established mechanism of hepatotoxicity.^59^ Analysis of patients treated with pyrimidine analogues has shown depleted mitochondrial and chromosomal DNA, leading to mitochondrial toxicity.^61,62^ Additionally, these chemicals showed high scores for thyroid hormone receptor (TR) antagonism (KE 24). The thyroid plays a critical role in maintaining liver lipid homeostasis by regulating the TR.^63−67^ Thus, these results suggest that the hepatotoxic effects of pyrimidine analogues with a ribose ring linked to a cytosine are due to the direct toxicity of hepatocytes through disrupting DNA replication or interfering with DNA in the mitochondria as well as altering lipid regulation from TR antagonism.

Cluster 26 consisted of 22 estrogens, a class of compounds used as oral contraceptives, to manage symptoms in menopause and to prevent osteoporosis. The chemicals in this cluster share a common 4-ring steroid backbone (Figure 2B). Most of these compounds are hepatotoxic and have manifested their hepatotoxicity in a variety of ways including cholestasis, hepatocellular carcinoma, and nonalcoholic fatty liver disease.^55,56,68−70^ The compounds had high KE scores for androgen receptor (AR) antagonism (KE 6) and progesterone receptor (PR) antagonism (KE 12). The AR controls critical liver functions and inhibition of AR leads to hepatotoxicity.^71−73^ Conversely, some studies have shown inhibition of PR to have a protective effect on hepatotoxicity.^74^ The opposing effects of these two pathways could account for the false positive results of this cluster. Three of the fourteen compounds in this cluster were not toxic to the liver. Notably, these three compounds, estriol (CAS 50-27-1), mestranol (CAS 72-33-3), and fulvestrant (CAS 129453-61-8), had higher PR antagonism (KE 12) scores than AR antagonism (KE 4). Based on these results, chemicals with the steroid backbone in this cluster, could disrupt liver functions as AR antagonists, although concurrent PR antagonism could mitigate these toxicity effects.

Other clusters, such as Clusters 16, 176, and 380, showed no clear mechanistic relationships but had modest scores for several KEs. Notably, Cluster 16 contains the most chemicals, such as phenothiazines which are antipsychotic drugs primarily used to treat schizophrenia and bipolar disorder and are a well-established class of hepatotoxicants.^75^ Most of these chemicals showed KE responses in DNA repair (KE 7) and TR antagonism (KE 24), KEs similar to Cluster 196.

### 3.5 Integration of Toxicokinetics for Hepatotoxicity Evaluations

Using in vitro responses to evaluate in vivo hepatotoxicity is difficult without the considering toxicokinetics (e.g., systemic exposure) of toxicants.^28,29^ For example, compounds with low in vivo oral bioavailability may not yield circulating concentrations that achieve the biological activities observed in in vitro assays. Conversely, compounds with high in vivo bioavailability are more likely to reach the concentrations at which they show in vitro activity and, thus, pose a higher risk to human health. Toxicokinetic modeling can be used to predict human systemic chemical availability by estimating the in vivo concentration of a compound after accounting for human metabolism and clearance, such as the concentration at steady-state (*C*_ss_). To show how this computational strategy can be integrated with chemical exposure data, we used a toxicokinetic model to predict blood plasma *C*_ss_ after repeated daily exposure of 1 mg/kg for target compounds. Comparing two chemicals with similar KE curves highlights the benefits of including toxicokinetics in AOP modeling of hepatotoxicity. Tamoxifen (CAS 10540-29-1) and fulvestrant (CAS 129453-61-8) are antiestrogens and breast cancer drugs. Tamoxifen and fulvestrant had multiple KE scores greater than 0.1, with 19 and 17, respectively (white and red areas in Figure 2A, the rows corresponding to tamoxifen and fulvestrant are annotated). The KE curves for these compounds are displayed in Figure 2C,D. While they have comparable KE curves, the bioavailability of these chemicals was predicted to be different. Tamoxifen has high bioavailability with a predicted *C*_ss_ of 22.00 mM, which is in agreement with experimental studies.^76^ Conversely, fulvestrant has low bioavailability as a predicted *C*_ss_ of 0.005 μM, also confirmed through experimental studies.^77^ Comparing the predicted bioavailability results of these chemicals to their KE scores gives potential inference into their discrepant hepatotoxicity outcomes (Figure 2C,D).^78^ Tamoxifen, a hepatotoxicant, has both high KE curves and in vivo bioavailability as *C*_ss_ (red shaded area in Figure 2C). Conversely, fulvestrant, which is not a hepatotoxicant, has low in vivo bioavailability as *C*_ss_ (red shaded area in Figure 2D), and most of its KE curves are out of the range for sufficient systemic exposure.

### 3.6 In Vivo Predictions of Hepatotoxicity

**Figure 3:**
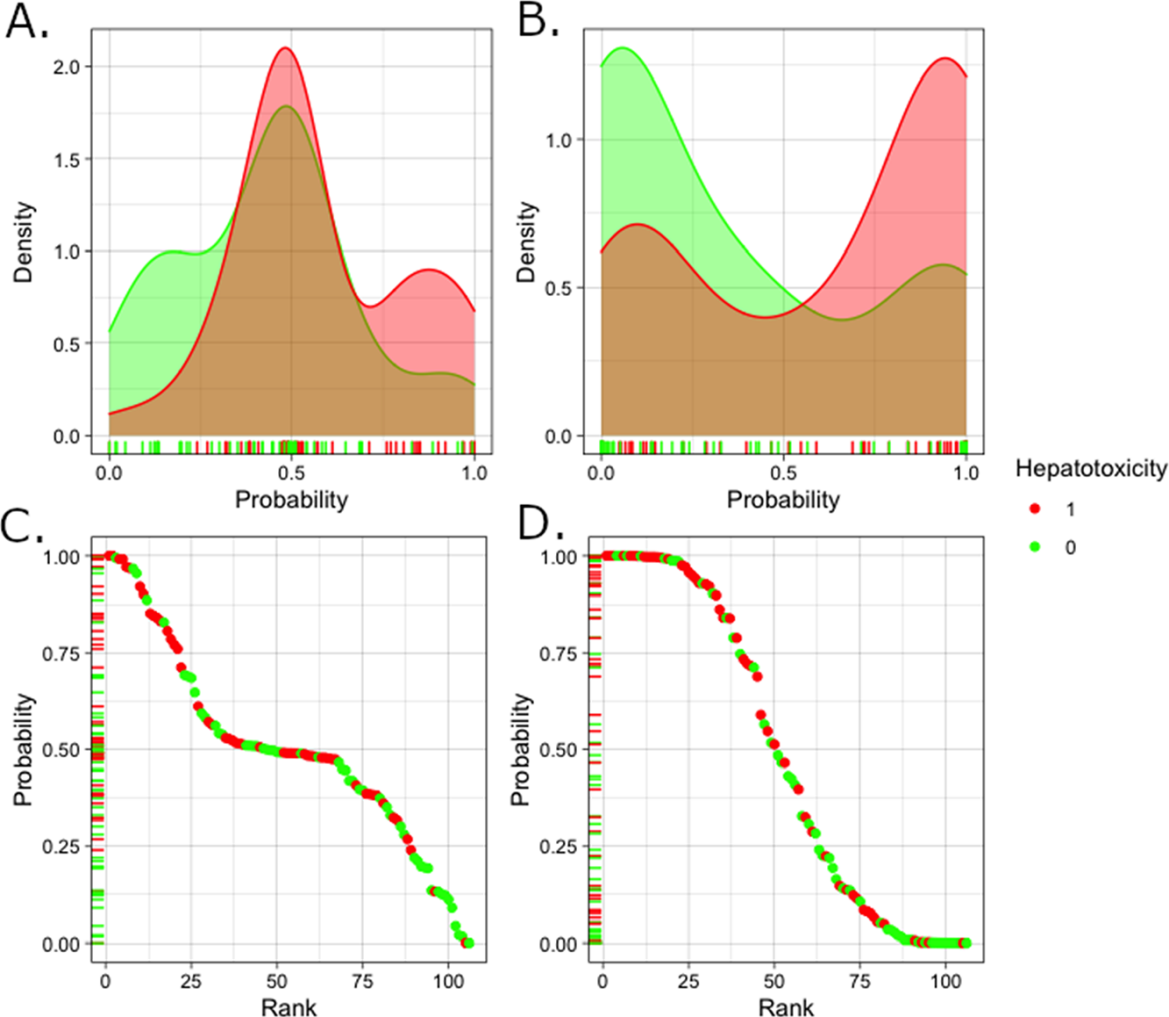
Performance of AOP Model 1 (using KE scores alone) and AOP Model 2 (using the combination of KE scores and bioavailability descriptors) shown as the leave-one-out cross validation results. (A) Distribution of probabilities for hepatotoxicants (red) and nonhepatotoxicants (green) of AOP Model 1. (B) Distribution of probabilities for hepatotoxicants (red) and nonhepatotoxicants (green) of AOP Model 2. (C) Chemicals ranked by probability as the outputs of AOP Model 1. (D) Chemicals ranked by probability as the outputs of AOP Model 2.

In order to screen large libraries of chemicals for hepatoxicity, automated methods that used supervised learning techniques were needed. To evaluate the potential of KE scores to be combined into a hepatoxicity model, we used the KE scores of chemicals to train a logistic regression model (Model 1) to directly predict hepatotoxicity in vivo. However, as shown above, the bioavailability of chemicals in vivo is also crucial to the success of using in vitro assays to predict in vivo endpoints.^79^ To this end, we also trained another logistic regression model using KE scores along with chemical-specific toxicokinetics information (Model 2). Bioavailability information used for modeling included *C*_ss_ values obtained through toxicokinetic modeling and molecular descriptors of Lipinski’s rule of five (see Methods). Of the ToxCast/Tox21 chemicals with hepatoxicity data, more chemicals were labeled as hepatotoxicants than nonhepatotoxicants (1200 of 1762 chemicals). To create a balanced dataset for logistic regression modeling, we randomly selected hepatotoxicants equal to the number of nonhepatotoxicants. The random undersampling method was used in several of our previous studies to balance the training data classifications.^26,27,80,81^ This final set of chemicals, consisting of 106 chemicals with toxicokinetic data (53 hepatotoxicants and 53 nonhepatotoxicants), was used to train both Model 1 and Model 2. This procedure involved leave-one-out validation by splitting the hepatotoxicity dataset where all but one chemical was used for model training and the remaining chemical was used as the test set and was repeated until each chemical was used for prediction. The prediction results of all compounds were averaged and used to evaluate model performance. The logistic regression model output for a chemical has a hepatotoxicity probability value ranging from 0 to 1 and chemicals with probability greater than 0.5 are classified as hepatotoxicants. The predictive performance of these models is summarized in Table 2 and the distribution of hepatotoxicity probabilities of hepatotoxic and nonhepatotoxic chemicals are shown in Figure 3.

**Figure 4:**
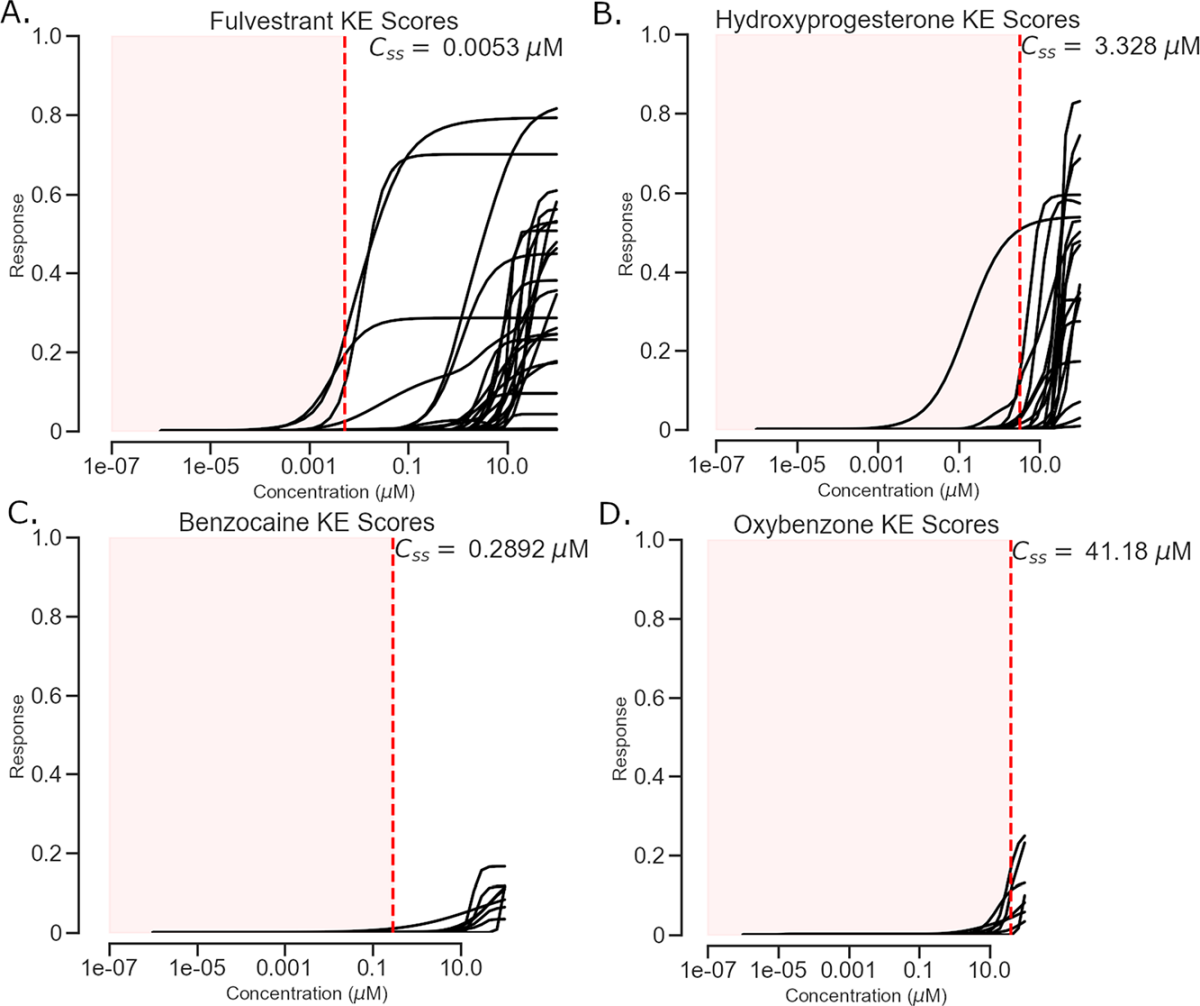
False positive compounds of AOP Model 1 corrected by AOP Model 2. Each panel shows a chemical’s KE curves along with potentially realized in vivo activities highlighted in the left red area. (A) Fulvestrant (CAS 129453-61-8). (B) Hydroxyprogesterone (CAS 630-56-8). (C) Benzocaine (CAS 94-09-7). (D) Oxybenzone (CAS 131-57-7).

The overall accuracy of Model 1 only using KE scores was 58%. This model predicted 51% of the hepatotoxic chemicals correctly (i.e., 51% recall). Integration of chemical bioavailability information improved the model performance by increasing accuracy from 58% (Model 1) to 67% (Model 2). The ability to detect hepatotoxic chemicals (i.e., recall) improved from 51% (Model 1) to 64% (Model 2), which is sufficiently aligned with current state-of-the-art methods of predicting hepatotoxicity. The advantage of including bioavailability information in the modeling process can be seen when observing the distribution of hepatotoxicity probabilities of hepatotoxicants and nonhepatotoxicants for Models 1 and 2 (Figure 3). Model 1 predicted 45 chemicals incorrectly, and 20 of them were correctly predicted after incorporating bioavailability information into the modeling process (Model 2). Nine of these chemicals are not hepatotoxicants, which the Model 1 using KE scores alone incorrectly predicted as hepatotoxicants. The reason is that these chemicals had relatively high KE scores. However, these nine chemicals had low estimated bioavailability with *C*_ss_ values that ranged from 0.005 to 83.11 μM. These values were much lower than the average *C*_ss_ value for the ToxCast/Tox21 chemicals as 1010 μM. For example, chemicals fulvestrant (CAS 129453-61-8), hydroxyprogesterone (CAS 630-56-8), benzocaine (CAS 94-09-7), and oxybenzone (CAS 131-57-7) are not hepatotoxicants and had KE curves out of their bioavailability ranges shown as *C*_ss_ (Figure 4). These results suggest that the use of KE scores alone (Model 1) is not sufficient for in vivo hepatotoxicity evaluation but inclusion of the toxicokinetic considerations can improve the applicability of models for predicting in vivo hepatotoxicity (Model 2). Integrating more chemical information, e.g., extra chemical descriptors, and analyzing their contributions to hepatotoxicity may further improve the model performance.

The benefit of adding chemical information was seen by performing a *t* test comparing the distributions of hepatotoxic and nonhepatotoxic compounds for each predictor variable in Model 2 (SupplementalFile.xlsx). The top predictor variables were either Lipinski descriptors (e.g., number of ring structures) or KE scores related to metabolism (e.g., CYP upregulation), indicating the importance of metabolic information. Integrating more chemical information, e.g., extra chemical descriptors, and analyzing their contributions to hepatotoxicity may further improve the model performance. We expect to perform this analysis after extending the current training data by including more chemicals.

The future of chemical toxicity testing is directed toward developing high-throughput, mechanism-based nonanimal approaches such as AOP models. Complex toxicity endpoints such as hepatotoxicity remain a challenge due to the diversity and complexity of the underlying toxicity mechanisms. In this work, we fill this gap in AOP modeling by presenting a new computational strategy that integrates concentration-dependent toxicity data and relevant toxicokinetics. Using hepatotoxicity as a proof-of-concept endpoint, we show that this method will identify and group HTS assays that measure key biological targets and processes relevant to hepatotoxicity. The KE scores generated by this computational modeling can be used to inform the mechanisms (i.e., the AOPs) causing the hepatotoxicity seen in certain chemical classes of hepatotoxicants. Additionally, KE curves allow for toxicity assessment across a range of concentrations and can be combined with toxicokinetic modeling to assess chemical toxicity at actual in vivo concentrations. Furthermore, we showed that logistic regression hepatotoxicity modeling integrating KE scores and bioavailability information can directly predict in vivo hepatotoxicity. Notably, this study is limited to the KE assays (i.e., ToxCast/Tox21 assays) selected by profiling the hepatotoxicity dataset. Therefore, the KEs identified in this study do not cover most hepatotoxicity AOPs reported (e.g., those on AOP-WiKi). In future studies, we can also use the AOP information as extra data to guide the selection of assays and relevant KEs. This research effort can evaluate the existing hepatotoxicity AOPs for their ability to identify new hepatotoxicants. This study showed that the use of public bioassay data and toxicokinetics results achieve acceptable prediction accuracy for hepatotoxicity evaluations, which are similar or superior to existing hepatotoxicity models.^79,81^ Additionally, this study was limited to KE scores generated from a relatively small dataset, including conservative read-across results. Incorporating larger datasets and performing experimental testing to fill the missing data of selected KE assays can greatly improve the model predictions, which have been validated in our previous modeling studies of chemical toxicities.^24,82^ The logistic regression approach was used for modeling due to the relatively small training set. Larger assay datasets for model development could allow for advanced machine-learning techniques to be used to construct the models and resulting in higher prediction accuracies. Lastly, to confirm the model predictions, in vivo experimental testing of predicted hepatotoxic compounds is needed to validate the models. This method advances moderncomputational toxicology by establishing an end-to-end workflow to evaluate chemical toxicity for complex toxicity endpoints.

## Supporting Information Available

KE classifications of the ToxCast/Tox21 assays along with descriptions and statistical association with hepatotoxicity data; distributions of the 157 ToxCast/Tox21 assays among the 52 KEs; area under the ROC curve scores for all KE models when predicting in vivo hepatotoxicity; results of the *T* test comparing the distributions of hepatotoxic and nonhepatotoxic compounds for each predictor variable (PDF)
Table showing features and p values (XLSX)
