# Supplementary material for: Integrating Concentration-Dependent Toxicity Data and Toxicokinetics To Inform Hepatotoxicity Response Pathways
Source: Environ Sci Technol. 2023 Aug 11;57(33):12291–301. doi: 10.1021/acs.est.3c02792 (PMC10448720; doi:10.1021/acs.est.3c02792)
Supplement: Supplementary file 1 — es3c02792_si_001.pdf [file es3c02792_si_001.pdf]

# Supporting Information

## Integrating concentration-dependent toxicity data and toxicokinetics to inform hepatotoxicity response pathways

*Daniel P. Russo*[1], *Lauren M. Aleksunes*[2], *Katy Goyak*[3], *Hua Qian*[3], *and Hao Zhu*[1*]
[1]Department of Chemistry and Biochemistry, Rowan University, Glassboro, New Jersey, 08028, USA;
[2]Department of Pharmacology and Toxicology, Ernest Mario School of Pharmacy, Rutgers University, Piscataway, New Jersey, 08854, USA;
[3]ExxonMobil Biomedical Sciences, Inc., Annandale, New Jersey, 08801, USA;

### Summary:

1 file (SupplementalFile.xlsx), including 4 tables.
